# DNAJA3/Tid1 Is Required for Mitochondrial DNA Maintenance and Regulates Migration and Invasion of Human Gastric Cancer Cells

**DOI:** 10.3390/cancers12113463

**Published:** 2020-11-20

**Authors:** Sheng-Fan Wang, Kuo-Hung Huang, Wei-Chuan Tseng, Jeng-Fan Lo, Anna Fen-Yau Li, Wen-Liang Fang, Chian-Feng Chen, Tien-Shun Yeh, Yuh-Lih Chang, Yueh-Ching Chou, Hung-Hsu Hung, Hsin-Chen Lee

**Affiliations:** 1 Department of Pharmacy, Taipei Veterans General Hospital, Taipei 112, Taiwan; sfwang5@vghtpe.gov.tw (S.-F.W.); ylchang@vghtpe.gov.tw (Y.-L.C.); ycchou@vghtpe.gov.tw (Y.-C.C.); 2 School of Pharmacy, Taipei Medical University, Taipei 110, Taiwan; 3 Department and Institute of Pharmacology, School of Medicine, National Yang-Ming University, Taipei 112, Taiwan; weichuan@gm.ym.edu.tw (W.-C.T.); jflo@ym.edu.tw (J.-F.L.); 4 School of Medicine, National Yang-Ming University, Taipei 112, Taiwan; khhuang@vghtpe.gov.tw (K.-H.H.); fyli@vghtpe.gov.tw (A.F.-Y.L.); wlfang@vghtpe.gov.tw (W.-L.F.); 5 Department of Surgery, Division of General Surgery, Taipei Veterans General Hospital, Taipei 112, Taiwan; 6 Department of Dentistry, School of Dentistry, National Yang-Ming University, Taipei 112, Taiwan; 7 Institute of Oral Biology, National Yang-Ming University, Taipei 112, Taiwan; 8 Cancer Progression Research Center, National Yang-Ming University, Taipei 112, Taiwan; cfchen@ym.edu.tw; 9 Department of Pathology, Taipei Veterans General Hospital, Taipei 112, Taiwan; 10 Institute of Anatomy and Cell Biology, School of Medicine, National Yang-Ming University, Taipei 112, Taiwan; tsyeh@ym.edu.tw; 11 Faculty of Pharmacy, National Yang-Ming University, Taipei 112, Taiwan; 12 School of Medicine, Faculty of Medicine, National Yang-Ming University, Taipei 112, Taiwan; 13 Department of Medicine, Division of Gastroenterology, Cheng Hsin General Hospital, Taipei 112, Taiwan

**Keywords:** gastric cancer, Tid1, mitochondria, cancer progression, galectin-7, MMP-9

## Abstract

**Simple Summary:**

Mitochondrial stress responses play a key role in cancer progression. Tid1, a mitochondrial co-chaperone, has been investigated and may act as a tumor suppressor in several cancers. However, the role of Tid1 in gastric cancers has not been clearly identified. Herein, we uncovered the clinical effects of Tid1 expression in gastric cancer patients. Moreover, we dissected the possible regulatory mechanism of Tid1 for cancer progression in gastric cancer cells. Our results demonstrated that Tid1 might regulate cell migration and invasion of gastric cancers. Tid1 may also be a key player for mitochondrial DNA maintenance in gastric cancer cells. We also found that the Tid1-galectin-7-MMP-9 axis might be one of the malignant regulatory mechanisms for gastric cancer metastasis and provide a potential target for gastric cancer treatment.

**Abstract:**

Background: Gastric cancer is a common health issue. Deregulated cellular energetics is regarded as a cancer hallmark and mitochondrial dysfunction might contribute to cancer progression. Tid1, a mitochondrial co-chaperone, may play a role as a tumor suppressor in various cancers, but the role of Tid1 in gastric cancers remains under investigated. Methods: The clinical TCGA online database and immunohistochemical staining for Tid1 expression in tumor samples of gastric cancer patients were analyzed. Tid1 knockdown by siRNA was applied to investigate the role of Tid1 in gastric cancer cells. Results: Low Tid1 protein-expressing gastric cancer patients had a poorer prognosis and higher lymph node invasion than high Tid1-expressing patients. Knockdown of Tid1 did not increase cell proliferation, colony/tumor sphere formation, or chemotherapy resistance in gastric cancer cells. However, Tid1 knockdown increased cell migration and invasion. Moreover, Tid1 knockdown reduced the mtDNA copy number of gastric cancer cells. In addition, the Tid1-galectin-7-MMP-9 axis might be associated with Tid1 knockdown–induced cell migration and invasion of gastric cancer cells. Conclusions: Tid1 is required for mtDNA maintenance and regulates migration and invasion of gastric cancer cells. Tid1 deletion may be a poor prognostic factor in gastric cancers and could be further investigated for development of gastric cancer treatments.

## 1. Introduction

Despite a recent and gradual decline in its incidence rate, gastric cancer remains a common cancer globally [1,2,3]. Eastern Asia has a high incidence of gastric cancer [2], whereas the incidence rates in Western countries are relatively low [2,3]. Environmental factors have been well studied to explain the regional difference in the incidence of gastric cancer [2,4]. The most effective treatment in gastric cancer is surgery or endoscopic resection, except in cases of late-stage cancer or an unresectable condition. Chemotherapy is an effective treatment choice for patients with adjuvant treatment or advanced-stage cancer and can effectively palliate the symptoms of gastric cancer and improve survival [5,6]. Despite advances in gastric cancer treatment, gastric cancer remains the 3rd leading cause of cancer deaths globally [2]. At present, due to the lack of effective screening for the early detection of gastric cancer, and the mild symptoms usually exhibited by these patients, most are initially diagnosed with advanced gastric cancer [7,8]. Moreover, unresectable advanced gastric cancers have a poor prognosis, with a median overall survival time of around 1 year [9]. Hence, understanding the regulation of gastric cancer progression is an important issue.

Mitochondria are intracellular organelles that are not only involved in cellular metabolism for generating adenosine triphosphate (ATP) and reactive oxygen species (ROS) byproducts via oxidative phosphorylation (OXPHOS), but also regulate apoptosis, cell differentiation, the cell cycle, and cell growth [10]. Deregulated cellular energetics is a cancer hallmark [11]. In cancer cells, the Warburg effect has been proposed as a characteristic of cancer cells that prefer using glycolysis than mitochondrial OXPHOS for glucose metabolism even under conditions of abundant oxygen [12]. Mitochondrial stress responses have thus been proposed as attractive targets for cancer treatment [13]. Mitochondria carry multiple copies of mitochondrial DNA (mtDNA), which is responsible for 13 subunits of OXPHOS [14]. In most gastric cancer, the mtDNA copy number is decreased and is associated with cancer progression [15,16,17]. Moreover, these mtDNA alterations and mitochondrial dysfunction might contribute to gastric cancer progression [18]. Hence, understanding the regulation of mtDNA alteration and mitochondrial hemostasis could assist the development of novel gastric cancer treatments.

Tumorous imaginal disc 1 (Tid1), also called DnaJ homolog subfamily A member 3, mitochondrial (DNAJA3), belongs to the heat shock protein (Hsp) 40 family. Tid1 is a co-chaperone that provokes ATPase activity of Hsp70, a molecular chaperone during protein refolding and transport, and provides the ability to adapt to environmental stress [19,20]. Two Tid1 mRNA splicing forms have been identified and produce two splice variants (Tid1-L and Tid1-S) of 43 and 40 kDa in cytosol, which are processed following import into the mitochondrial matrix [20,21]. Mitochondrial Tid1 is responsible for maintaining mtDNA integrity and mitochondrial membrane potential, whereas cytosolic Tid1 interacts with Hsp 70 and executes cell signaling pathways [20,21,22]. Tid1 is involved in growth, proliferation, differentiation, senescence, survival, apoptosis, and movement and plays important roles in development of embryo and skeletal muscle [23,24,25,26,27,28,29]. Evidence suggests that high Tid1-expressing head and neck cancer patients have better survival rate, lower malignancy, and lower recurrence than low Tid1-expressing patients [30]. In addition, lower expression of Tid1 has a relatively higher risk of malignant characteristics, such as advanced stages of cancer, larger tumor lesions, and lymphovascular invasion in breast cancer patients [31]. Moreover, both gene expressions of Tid1-L and Tid1-S were reduced in tumors of non-small cell lung cancer (NSCLC) patients. Low Tid1-L/high epidermal growth factor receptor (EGFR) expression is related to poor overall survival in NSCLC patients [32]. Conversely, high Tid1-expression contributes to cancer progression in colorectal cancers [33]. Tid1-S expression was found to be highly expressed in stage IV NSCLC patients, and high Tid1-S/EGFR levels were correlated with lymph node metastasis and poor overall survival of NSCLC patients [34]. These lines of evidence suggest that the role of Tid1 in cancer progression might be dependent on the cancer type and the variants of Tid1. However, the role of Tid1 in gastric cancer remains unclear. Moreover, the role of Tid1 in mitochondria homeostasis in gastric cancer has not been identified, although we previously identified that mtDNA loss and mitochondrial dysfunction might be involved in gastric cancer progression. In the present study, we dissected the effects of Tid1 knockdown on mitochondrial homeostasis and evaluated the role of Tid1 expression in gastric cancers.

## 2. Results

### 2.1. Low Tid1 Protein-Expressing Gastric Cancer Patients Have Poorer Prognosis than High Tid1-Expressing Patients

**Figure 1:**
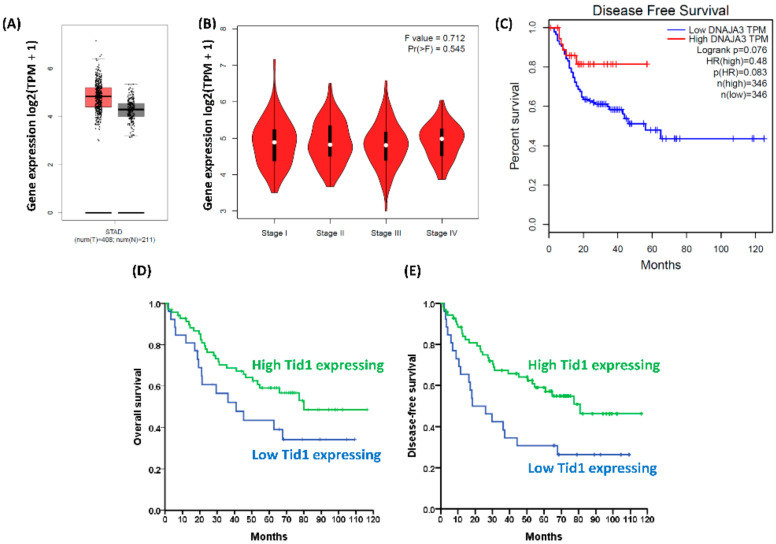
The expression of Tid1 and its clinical effects in gastric cancers. (**A**–**C**) Using the TCGA database and the GTEx project, the boxplot, stage plot, and Kaplan–Meier plot were analyzed using the GEPIA online database and software (http://gepia.cancer-pku.cn/). (**A**) The gene expression of Tid1 in normal tissues (gray box) and tumor tissues (red box) was analyzed by box-plot. STAD: stomach adenocarcinoma, T (tumor) number: 408, N (normal) number: 211; |Log_2_FC| cutoff: 1; *p*-value cutoff: 0.01. (**B**) The association between gastric cancer stages and Tid1 gene expression was analyzed by stage-plot. *F* value = 0.712; Pr (>F) = 0.545. (**C**) The Kaplan–Meier survival analysis for Tid1 expression and DFS in gastric cancer patients. Log-rank *p* = 0.076. (**D**,**E**) Using immunohistochemical (IHC) staining for Tid1 protein expression in gastric cancer samples, we compared survival between gastric cancer patients with high and low Tid1 protein expression. (**D**) Gastric cancer patients with high Tid1 expression had a trend of better OS rates than those with low Tid1 expression, *p* = 0.082 (log rank test). (**E**) Gastric cancer patients with high Tid1 expression had a better DFS rate than those with low Tid1 expression, *p* = 0.008 (log rank test).

To evaluate the role of Tid1 in gastric cancer, we evaluated the gene expression of Tid1 in gastric cancer patients. Using the GEPIA online database, which is based on the TCGA database and GTEx project, we found that gene expression of Tid1 is higher in tumors than in the corresponding normal tissues in gastric cancer patients (Figure 1A). However, Tid1 gene expression might not be associated with the stages of gastric cancer (Figure 1B). The low Tid1 gene-expressing gastric cancers showed a trend of lower disease-free survival (DFS) rate than high Tid1 gene-expressing gastric cancer patients, although this difference was not statistically significant difference (Figure 1C). We further evaluated the clinical impact of Tid1 protein expression in the tumors of gastric cancer patients using immunohistochemical staining. Of the 100 gastric cancer patients, the tumors of 70 (70%) showed high Tid1 expression and the tumors of 30 (30%) showed low Tid1 expression (Table 1). With respect to the gross and microscopic appearance of the gastric cancer specimens with high or low Tid1 expression, there were no significant differences between these gross and histological characteristics. Based on American Joint Committee on Cancer (AJCC) tumor-node-metastasis (TNM) staging [35], less lymph node metastasis (*p* = 0.041) was observed in gastric cancer specimens with high Tid1 expression. The 5 year overall survival (OS) rates of gastric cancer patients with high and low Tid1 expression were 58.5 and 43.3%, respectively (*p* = 0.082, Figure 1D). The 5 year DFS rates of gastric cancer patients with high and low Tid1 expression were 57.1 and 36.7%, respectively (*p* = 0.008, Figure 1E). The clinical data show that high Tid1 protein expression represents less aggressive tumor behavior compared to low Tid1 expression.

### 2.2. Tid1 Does Not Affect Cell Proliferation, Colony Formation, Tumor Sphere Formation, or Chemotherapy Resistance of Human Gastric Cancer Cells

**Figure 2:**
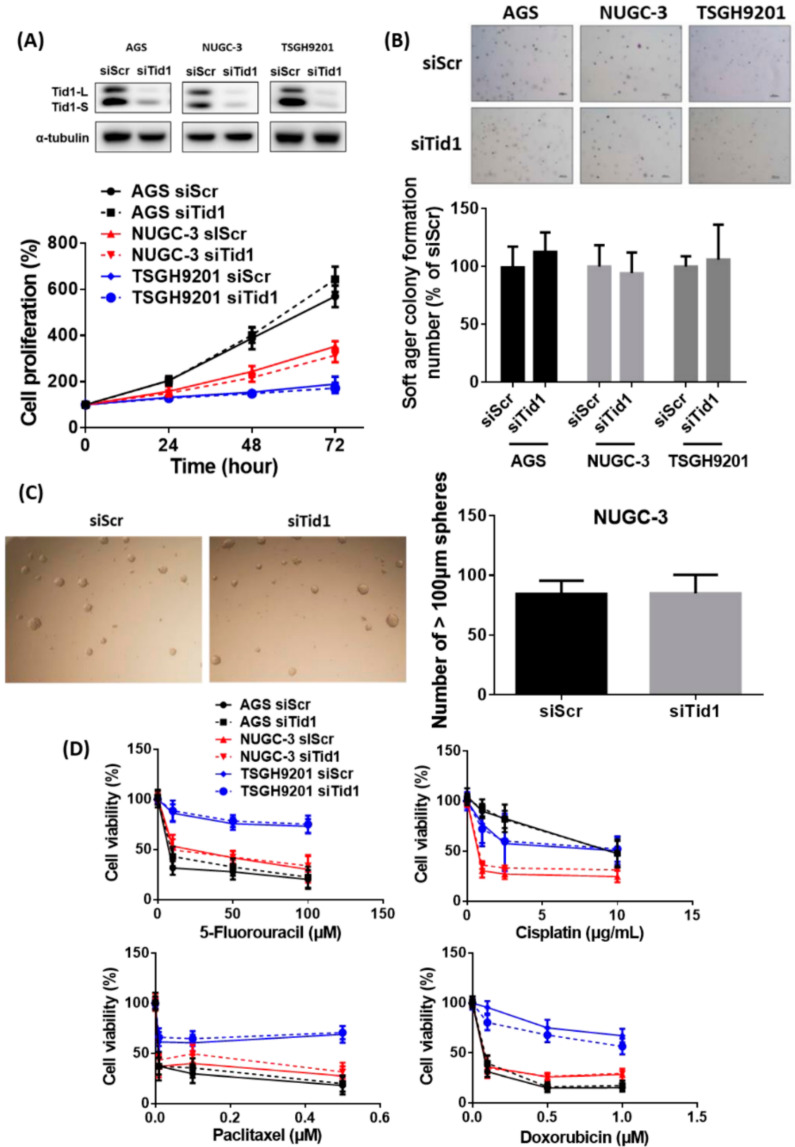
Effect of Tid1 knockdown on cell proliferation, colony formation, tumor sphere formation, and chemotherapy sensitivity of gastric cancer cells. (**A**) The effect of Tid1 knockdown on cell proliferation was evaluated by the sulforhodamine B (SRB) assay. After knockdown of Tid1 by siRNA, the gastric cancer cells were reseeded and cell proliferation was evaluated for 3 days. (**B**) The effect of Tid1 knockdown on colony formation was evaluated using a soft agar colony formation assay. After knockdown of Tid1 by siRNA, the gastric cancer cells were reseeded and colony formation was evaluated for 30 days. (**C**) The effect of Tid1 knockdown on tumor sphere formation was evaluated using a tumor sphere formation assay. After knockdown of Tid1 by siRNA, the NUGC-3 gastric cancer cells were reseeded and sphere formation was evaluated for 10 days. (**D**) The chemotherapy sensitivity was evaluated using a SRB assay. After knockdown of Tid1 by siRNA, the gastric cancer cells were reseeded and treated with different chemotherapy agents for 2 days. The efficiency of Tid1 knockdown was confirmed by Western blot. Data are presented as mean ± SD of at least three independent experiments. *p* < 0.05, compared with the siRNA for scramble (siScr) control group.

To evaluate the role of Tid1 in gastric cancer progression, we used siRNA to knockdown Tid1 expression in human gastric cancer cells, AGS, NUGC-3, and TSGH9201. No obvious cell death was observed during the siRNA transfection. We found that knockdown of Tid1 does not significantly affect cell proliferation (Figure 2A) and colony formation (Figure 2B) in these cell lines. Moreover, knockdown of Tid1 did not change the ability of tumor sphere formation of NUGC-3 cells (Figure 2C). Previous evidence showed that knockdown of Tid1 contributes to resistance of exogenous stress, particularly chemotherapeutic agents such as cisplatin and mitomycin C [36]. However, our results revealed that Tid1 knockdown does not interfere with sensitivities of chemotherapeutic agents such as 5-fluorouracil, cisplatin, paclitaxel, and doxorubicin in these cell lines (Figure 2D). These results suggest that Tid1 might not be involved in the regulation of cell proliferation, colony formation, tumor sphere formation, or chemotherapy resistance in gastric cancer cells. Original uncropped western blots images were provided in Figure S1.

### 2.3. Tid1 Knockdown Increases Cell Migration and Invasion of Gastric Cancer Cells

**Figure 3:**
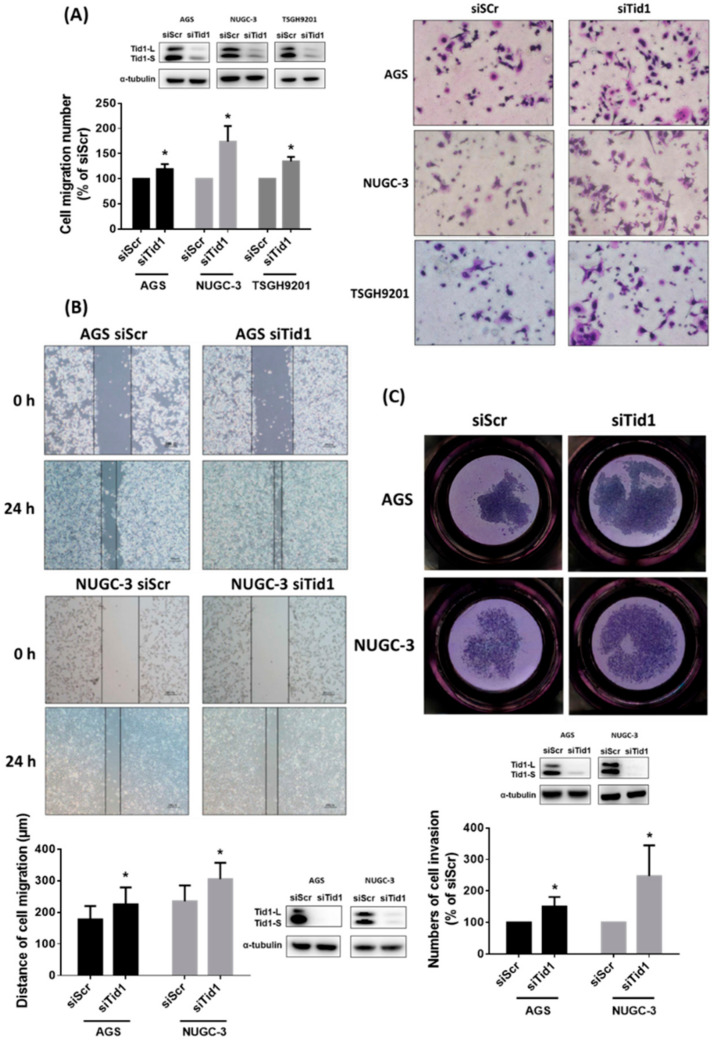
Effect of Tid1 knockdown on cell migration and invasion of gastric cancer cells. (**A**,**B**) The effect of Tid1 knockdown on cell migration was evaluated by (**A**) the transwell migration assay and (**B**) the wound healing assay. After knockdown of Tid1 by siRNA, the gastric cancer cells were reseeded and cell migration was evaluated by the transwell assay (AGS for 8 h, NUGC-3 for 16 h, and TSGH9201 for 24 h) and the 24 h wound healing assay. (**C**) The effect of Tid1 knockdown on cell invasion was evaluated by the transwell invasion assay. After knockdown of Tid1 by siRNA, the gastric cancer cells were reseeded and cell invasion was evaluated by the transwell invasion assay for 48 h. The efficiency of Tid1 knockdown was confirmed by Western blot. Data are shown as the mean ± SD of at least three independent experiments. *, *p* < 0.05, compared with the siRNA for scramble (siScr) control group.

We further evaluated the role of Tid1 in migration and invasion of human gastric cancer cells. Initially, we perfumed evaluation at different time points using a transwell migration assay for three individual gastric cancer cell lines (migration for 8 h in AGS cells; migration for 16 h in NUGC-3 cells; migration for 24 h in TSGH9201 cells) based on their characteristics. This showed that the knockdown of Tid1 can significantly increase the ability of transwell migration in gastric cancer cells (Figure 3A). Original uncropped western blots images were provided in Figure S2. Because TSGH9201 gastric cancer cells have little ability with respect to wound healing migration and transwell invasion, we performed wound healing migration and transwell invasion assays in the AGS and NUGC-3 gastric cancer cells. The results of the wound healing assay showed that Tid1 knockdown can increase the ability of wound healing migration (Figure 3B). Original uncropped western blots images were provided in Figure S3. Moreover, Tid1 knockdown also increased the ability of transwell invasion (Figure 3C). Original uncropped western blots images were provided in Figure S4. These results revealed that Tid1 knockdown might contribute to enhanced abilities of cell migration and invasion in gastric cancer cells.

### 2.4. Tid1 Is Required for mtDNA Maintenance, but Tid1 Knockdown Might Not Consistently Interfere with Mitochondrial Gene Expression, Content, and Respiratory Function in Gastric Cancer Cells

**Figure 4:**
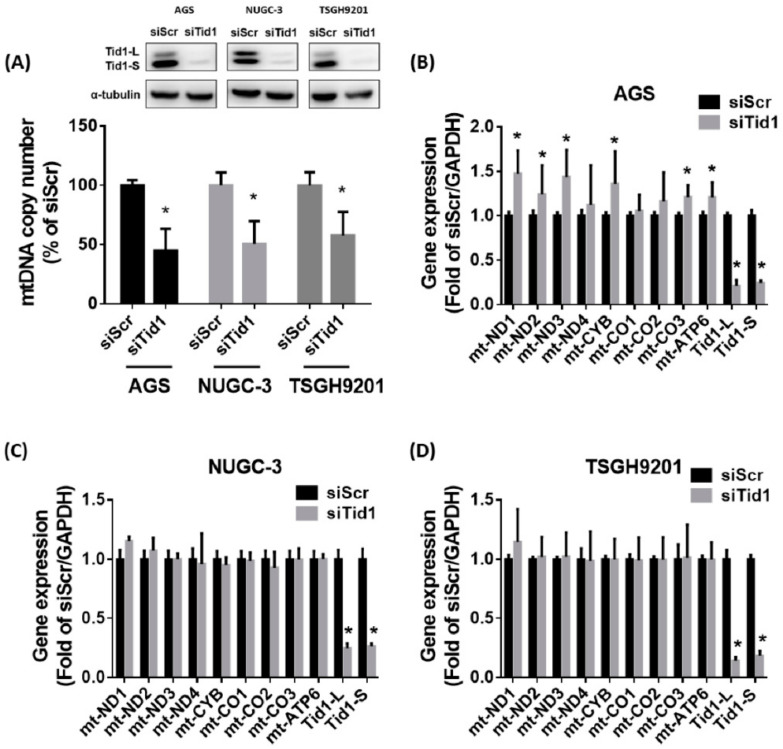
Effect of Tid1 knockdown on mtDNA copy number and mitochondrial gene expression of gastric cancer cells. (**A**) The effect of Tid1 knockdown on mtDNA copy number was evaluated by real-time PCR. (**B**–**D**) The effect of Tid1 knockdown on mitochondrial gene expression was evaluated by reverse transcription (RT)-real-time PCR in the (**B**) AGS, (**C**) NUGC-3, and (**D**) TSGH 9201 gastric cancer cells. The efficiency of Tid1 knockdown was confirmed by Western blot or RT-real-time PCR. Data are shown as the mean ± SD of at least three independent experiments. *, *p* < 0.05, compared with the siRNA for scramble (siScr) control group.

**Figure 5:**
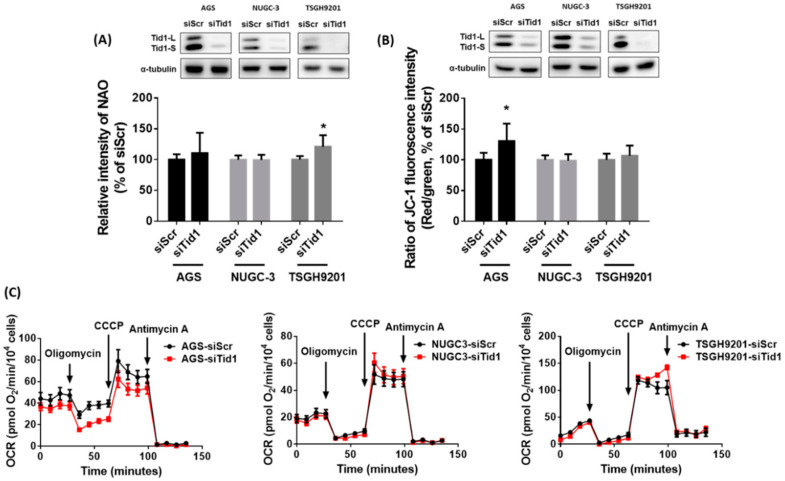
Effects of Tid1 knockdown on mitochondrial mass, mitochondrial membrane potential, and mitochondrial oxygen consumption rate (OCR) of gastric cancer cells. (**A**) The effect of Tid1 knockdown on mitochondrial mass was evaluated by flow cytometry with nonyl acridine orange (NAO) staining. (**B**) The effect of Tid1 knockdown on mitochondrial membrane potential was evaluated by flow cytometry with JC-1 staining. (**C**) The effect of Tid1 knockdown on mitochondrial OCR was determined by Seahorse XF^e^ Analyzer. After knockdown of Tid1 by siRNA, the gastric cancer cells were reseeded and mitochondrial OCR was evaluated until cells were attached (overnight). The efficiency of Tid1 knockdown was confirmed by Western blot. Data are shown as the mean ± SD of two or three independent experiments. *, *p* < 0.05, compared with the siRNA for scramble (siScr) control group.

Previous evidence has shown that Tid1 might play an important role in mitochondria homeostasis [22]. In addition, mtDNA abnormalities were found in gastric cancer tissues [15,18]. We evaluated the effect of Tid1 knockdown on the mitochondria of human gastric cancer cells AGS, NUGC-3, and TSGH9201. We found that knockdown of Tid1 by siRNA significantly decreased the mtDNA copy number in these gastric cancer cells (Figure 4A). Knockdown of Tid1 did not decrease the transcript levels of mitochondrial genes in gastric cancer cells, whereas the transcript level was slightly increased in the AGS gastric cancer cells (Figure 4B–D). Original uncropped western blots images were provided in Figure S5. Furthermore, knockdown of Tid1 did not significantly affect the mitochondrial mass in gastric cancer cells, with the exception that it was slightly elevated in the TSGH9201 gastric cancer cells (Figure 5A). Original uncropped western blots images were provided in Figure S6. Moreover, knockdown of Tid1 in gastric cancer cells had similar mitochondrial membrane potential as that of control cells, with the exception of AGS gastric cancer cells, in which it increased (Figure 5B). Original uncropped western blots images were provided in Figure S7. Tid1 knockdown did not consistently affect the basal and maximal mitochondrial oxygen consumption rate (Figure 5C). These results suggest that Tid1 is required for mtDNA copy number maintenance, but might not consistently regulate mitochondrial gene expression, mitochondrial content, or respiratory function in gastric cancer cells.

### 2.5. Galectin-7-Matrix Metallopeptidase 9 (MMP-9) Axis Is Associated with the Tid1 Knockdown-Increased Cell Migration and Invasion of Gastric Cancer Cells

**Figure 6:**
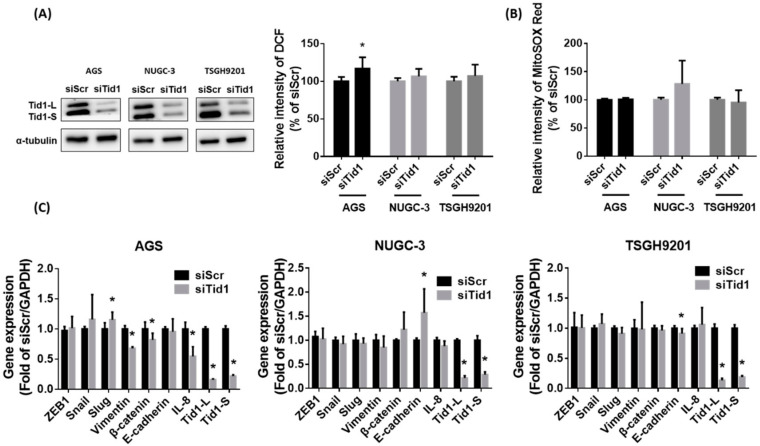
Effect of Tid1 knockdown on cellular, mitochondrial reactive oxygen species (ROS) and the gene expressions of epithelial mesenchymal transition (EMT) and interleukin 8 (IL-8) of gastric cancer cells. (**A**,**B**) The effect of Tid1 knockdown on cellular (**A**) and mitochondrial (**B**) ROS was evaluated by flow cytometry with dichlorodihydro-fluorescein diacetate (DCFH-dA) and MitoSOX Red staining, respectively. (**C**) The effect of Tid1 knockdown on EMT/ IL-8 gene expression was evaluated by RT-real-time PCR. The efficiency of Tid1 knockdown was confirmed by Western blot (**A**,**B**) or RT-realtime PCR (**C**). Data are shown as the mean ± SD of at least three independent experiments. *, *p* < 0.05, compared with the siRNA for scramble (siScr) control group.

It has been reported that ROS play an important role in the mitochondrial dysfunction-increased migration of gastric cancer cells [37,38]. In in vitro experiments, mitochondrial inhibitor-mediated mitochondrial dysfunction might contribute to chemoresistance and enhanced cell migration [38]. The ROS-mediated β5-integrin pathway is involved in mitochondrial dysfunction-increased cell migration [37]. In addition, mitochondrial dysfunction enhances cisplatin resistance in human gastric cancer cells through the ROS-activated integrated stress response [39]. However, our results revealed that knockdown of Tid1 did not significantly change the cellular and mitochondrial ROS levels, with the exception that cellular ROS were slightly increased in AGS gastric cancer cells (Figure 6A,B). Original uncropped western blots images were provided in Figure S8. These results suggest that ROS is not involved in the Tid1 knockdown-increased cell migration and invasion of human gastric cancer cells.

Some lines of evidence showed that epithelial to mesenchymal transition (EMT) and interleukin-8 (IL-8) might be involved in the Tid1 knockdown-increased metastasis in cancer cells [40,41]. Tid1 is involved in the mitochondrial translocation of p53Ψ, a unique p53 transcriptionally inactive isoform, and contributes to cell migration of lung cancer cells via EMT. An important characteristic of metastatic cells and mesenchymal-like features is that they tend to be related to greater cell migration and invasion [40,42]. In breast cancer patients, the level of IL-8, a member of the CXC chemokine family, was higher in tumor tissues than in normal tissues [43]. IL-8 also contributes to metastatic characteristics in breast cancer cells [44,45]. Evidence has shown that Tid1 depletion-mediated migratory potential of breast cancer cells was consistent with elevation of IL-8 [41]. However, our results showed that the gene expressions of EMT are not consistently increased (increased in Slug and decreased in vimentin and β-catenin for AGS cells; increased in E-cadherin for NUGC-3 cells; decreased in E-cadherin for TSGH9201 cells) by Tid1 knockdown in the AGS, NUGC-3, and TSGH9201 gastric cancer cells (Figure 6C). Moreover, the gene expression of IL-8 is not increased in the AGS, NUGC-3, and TSGH9201 gastric cancer cells, whereas IL-8 is decreased in the AGS cells (Figure 6C). These results suggest that EMT and IL-8 might not significantly contribute to the Tid1 knockdown-increased migration and invasion of gastric cancer cells.

**Figure 7:**
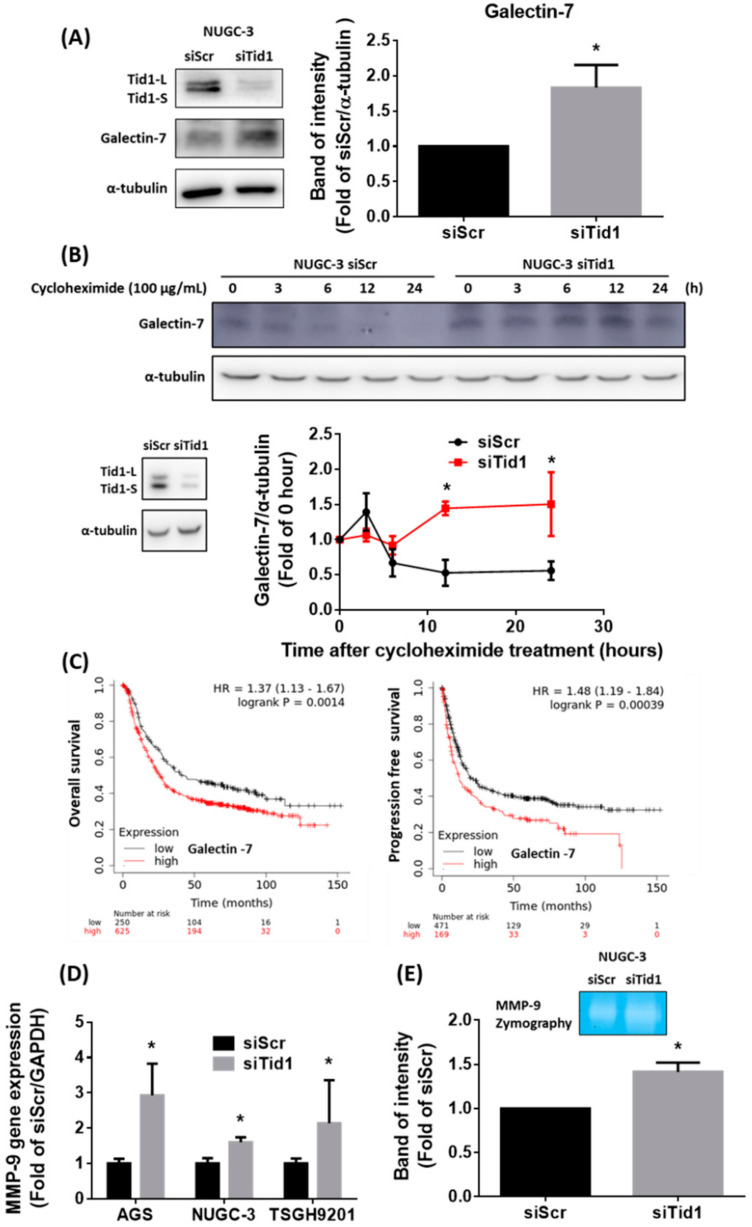
The Tid1-galectin-7-MMP-9 pathway might contribute to cell migration and invasion in NUGC-3 gastric cancer cells. (**A**) The effect of Tid1 knockdown on galectin-7 expression was determined by Western blot. (**B**) The effect of Tid1 knockdown on galectin-7 protein stability was determined by cycloheximide chase analysis. After knockdown of Tid1 by siRNA, the gastric cancer cells were reseeded and treated with cycloheximide for the indicated time periods. (**C**) Kaplan–Meier survival analyses were performed using the Kaplan–Meier plotter online database (http://kmplot.com/analysis/) [47]. The results showed the effect of galectin-7 gene expression on overall survival (OS, left) and progression-free survival (PFS, right) in gastric cancer patients. (**D**,**E**) The effects of Tid1 knockdown on gene expression and activity of MMP-9 were determined by (**D**) RT-real-time PCR and (**E**) gelatin zymography analysis. The efficiency of Tid1 knockdown was confirmed by Western blot and RT-real-time PCR. Data are shown as the mean ± SD of at least three independent experiments. *, *p* < 0.05, compared with the siRNA for scramble (siScr) control group.

Recent research has suggested that the galectin-7-TCF3-matrix metallopeptidase (MMP-9) axis is a key regulator for Tid1-suppressed metastasis in head and neck cancer cells [46]. To test this axis, we evaluated the effect of Tid1 knockdown on galectin-7 expression. Our results revealed that Tid1 knockdown can increase the protein expression of galectin-7 (Figure 7A). Original uncropped western blots images were provided in Figure S9. Moreover, we pre-treated with cycloheximide to block protein synthesis and found that knockdown of Tid1 increased the protein stability of galectin-7 by repressing its degradation (Figure 7B). Original uncropped western blots images were provided in Figure S10. Furthermore, using the Kaplan–Meier plotter online database, which is based on the GEO, EGA, and TCGA databases, we found that galectin-7 gene expression might be a poor prognosis factor in gastric cancer patients (Figure 7C). Original uncropped western blots images were provided in Figure S11. Importantly, we found that Tid1 knockdown elevated the MMP-9 gene expression of MMP-9 in the AGS, NUGC-3, and TSGH9201 gastric cancer cells (Figure 7D). Moreover, the activity of MMP-9 increased by Tid1 knockdown in the NUGC-3 gastric cancer cells (Figure 7E). Our results revealed that the galectin-7-MMP-9 pathway might be a regulatory mechanism for the Tid1 knockdown-increased cell migration and invasion of gastric cancer cells.

## 3. Discussion

In the present study, we clearly demonstrated that Tid1 deletion might be a poor prognostic factor for gastric cancers. Clinical evidence showed that low Tid1 protein-expressing gastric cancer patients have higher lymph node invasion and poorer prognosis than high Tid1 protein-expressing patients. We further evaluated the effects of Tid1 on the cancer progression in several gastric cancer cell lines. Although Tid1 knockdown could not increase cell proliferation, colony formation, tumor sphere formation or chemoresistance, the knockdown of Tid1 might increase the abilities of migration and invasion of gastric cancer cells. To explore the role of Tid1 in mitochondria homeostasis during gastric cancer progression, we evaluated the effect of Tid1 knockdown on mitochondrial homeostasis. However, the knockdown of Tid1 only decreased the copy number of mtDNA, and did not consistently affect the mitochondrial content, respiratory function, and ROS production. These results suggest that Tid1 is required for mtDNA maintenance, and also regulates cell migration and invasion of human gastric cancer cells.

The J domain is the highly conserved region in Tid1/*DNAJA3* and provides the binding ability with Hsp70 for protein folding, protein degradation, assembly/disassembly of multiprotein complexes, and protein translocation [48,49,50,51,52]. Hsp70-mediated ubiquitination and degradation of oncoproteins, such as epidermal growth factor receptor, HER2, and hypoxia-inducible factor-1α, might provide Tid1 with tumor suppressor ability [31,32,53]. Tid1-L and Tid1-S, two alternatively spliced variants, have different amino acid sequences in the C-terminal region. Tid1-L has been demonstrated to harbor high cytosolic stability and contribute to apoptosis induction, whereas Tid1-S prefers translocation to mitochondria and counteracts the apoptosis process [20]. In the present study, we applied knockdown to both Tid1-L and Tid1-S by specific siRNA against Tid1. Hence, the detailed regulatory roles of the two spliced variants of Tid1 for cancer progression remain under-investigated. These two Tid1 variant proteins might have different regulatory mechanisms for cancer progression [27,32]. To date, although the differential binding proteins of these variants have been identified, the functions of these client proteins largely remain to be investigated [46].

Previous evidence showed that mtDNA depletion occurs in most gastric cancers (55%) and around half of gastric cancer patients had somatic mutations in the D-loop, which is the key area of the mutated region and is responsible for the replication and transcription of mtDNA [15]. The proportion of mtDNA depletion was associated with the ulcerated, infiltrating (Borrmann type III) and diffusely thick (Borrmann type IV) types of gastric carcinomas, indicating that mtDNA depletion might contribute to gastric cancer progression. The induction of EMT and stemness ability might contribute to invasiveness and metastasis in MtDNA-depleted cancer cells [54]. MtDNA depletion elevated cancer stem cell characteristics in prostate cancer cells [55]. Mutant mitochondrial polymerase γ-mediated or ethidium bromide-treatment-mediated mtDNA loss might induce an invasive phenotype in cancer cells [56,57].

In the present study, we found that the mtDNA copy number was reduced with Tid1 knockdown, whereas the mtDNA gene transcription, mitochondrial mass, mitochondrial membrane potential, ROS, and mitochondrial OCR were not consistently changed by Tid1 knockdown in all three gastric cancer cell lines. We used NAO staining (recognizing the cardiolipin, which is an important phospholipid found almost exclusively in the inner mitochondrial membrane) for determination of mitochondrial content. Our results robustly demonstrated that the level of cardiolipin was not consistently affected by Tid1 knockdown. In addition, we found that the mitochondrial content might not be reduced by Tid1 knockdown using another MitoTracker™ Green FM staining assay. By comparison, the basal level of Tid1 in the TSGH9201 cells is relatively lower than that of the AGS and NUGC-3 gastric cancer cells. Moreover, the cell types and malignant grading of these cell lines vary (AGS: human gastric adenocarcinoma; NUGC-3: human metastatic gastric adenocarcinoma; TSGH9201: human metastatic signet ring cell gastric adenocarcinoma). This implies that mitochondrial effects of Tid1 knockdown might depend on specific cell types. Previous evidence has shown that chaperones and co-chaperones are involved in mitochondrial homeostasis. Tid1 can regulate mitochondrial homeostasis via DNA polymerase γ, dynamin-1-like protein, optic atrophy 1, and CR6-interacting factor 1 [58,59,60,61]. Importantly, it was noted that only prolonged silencing of Tid1 can significantly affect mtDNA gene expression and OCR [22]. Thus, our present results suggest that short-term knockdown of Tid1 by siRNA is required for mtDNA maintenance but is not sufficient to affect mitochondrial gene expression and respiratory function. In the present study, we found that the contribution of short-term Tid1 knockdown by siRNA to cancer progression might not be mediated by mitochondrial dysfunction.

Tid1 has also been found to be highly associated with cancer development. Many Hsp 40 family proteins are highly expressed in certain types of cancer [62,63,64,65]. Tid1 can act as a tumor suppressor by forming a complex with p53 and translocating to mitochondria for subsequent execution of the mitochondrial apoptosis pathway [27]. Moreover, Tid1 has an antitumor function via the reduction in the malignant activity of human epidermal growth factor receptor 2 (HER2) in cancer cells [66]. The relationship between Tid1 and cancer progression has been extensively investigated in studies of HER2, c-Met receptor tyrosine kinase (MetR), signal transducer and activator of transcription 5 b (STAT5b), Akt, adenomatous polyposis coli, and galectin-7 [30,31,46,67,68,69]. Our results might exclude the role of mitochondrial dysfunction in the Tid1 knockdown-mediated gastric cancer progression. In the present study, we identified that Tid1 knockdown increases the cell migration and invasion of gastric cancer cells. We also demonstrated that knockdown of Tid1 might contribute to cancer progression via the galectin-7-MMP-9 pathway, but not via mitochondrial dysfunction, ROS, EMT, and IL-8.

Galectin-7, one of the family of β-galactoside-binding proteins is responsible for epithelial homeostasis, cell adhesion, and migration [70]. Galectin-7 is able to form homodimers in the cytosol, mitochondria, and nucleus, but its function in the nucleus is still unknown. Galectin-7 has attracted significant attention as a diagnostic biomarker, tumor progression marker, and therapeutic target for cancer treatment [71,72,73,74]. Although galectin-7 might play an important role in cancer progression, the evidence of galectin-7 in gastric cancer progression is limited. Evidence showed that galectin-7 can be regulated by the DNA methylation-mediated epigenetic mechanism and might have a tumor suppression role in gastric cancer [75]. Our results suggest that high galectin-7 gene expression might be a poor prognostic factor for gastric cancer patients. The role of Tid1 in cancer progression continues to be debated, and the role of Tid1 in cancer may be distinctive [76]. It has been shown that the galectin-7-TCF3-MMP-9 pathway is involved in Tid1 knockdown mediated-cancer progression in head and neck cancers [46]. The present study further demonstrated that knockdown of Tid1 contributes to cell migration and invasion through the galectin-7-MMP-9 axis in gastric cancer cells. MMP-9 is a zinc-dependent protease that is responsible for degradation of components of the extracellular matrix, such as gelatin, and plays a critical role in cancer metastasis and progression [77]. Some lines of evidence have shown that MMP-9 expression might be involved in gastric cancer invasion and is a predictor of outcomes in metastatic gastric cancer patients [78,79]. Moreover, HER2 can promote gastric cancer malignancy by regulating MMP-9 [80]. Our present results demonstrate that Tid1 knockdown might contribute to migration and invasion in gastric cancer cells via the galectin-7-MMP-9 axis. This might provide a potential treatment target for low Tid1-expressing gastric cancers.

## 4. Materials and Methods

### 4.1. Clinical Gene Expression and Survival Analyses

Analysis of clinical RNA sequencing expression was performed using the GEPIA online database and software (http://gepia.cancer-pku.cn/) [81]. The clinical data were collected from the TCGA and the GTEx project. The gene expression of Tid1 in normal (gray box) and tumor (red box) tissues from stomach adenocarcinoma (STAD) was analyzed by box-plot. The relationship between Tid1 gene expression and cancer stages was analyzed using the pathological stage plot. The DFS was analyzed by Kaplan–Meier (KM) plot survival analyses (cutoff-high 10%). The analysis of galectin-7 gene expression was performed using the KM plotter online database and software (https://kmplot.com/analysis/) [47]. The *LGALS7* (galectin-7) gene (Affy ID/gene symbol 206400_at) was analyzed using an mRNA gene chip (Kaplan–Meier plotter online database, http://kmplot.com/analysis/) in gastric cancer patients for KM analysis.

### 4.2. Patient Sample Collection and Follow-Up

A total of 100 gastric cancer patients were enrolled at the Taipei Veterans General Hospital. These patients underwent curative resection for gastric cancer. The pre-operative evaluation, surgical methods, and collection of clinical data were described in a previous protocol [82]. Informed consent for sample collection was obtained from all patients before operation. The analysis of the tissue specimens was approved by the Institutional Review Board (IRB) of Taipei Veterans General Hospital (TPEVGH IRB No.:2019-06-004BC).

Gross features of the gastric tumor sample were based on tumor size, tumor location, and the Borrmann classification. The pathological features were analyzed according to tumor cell differentiation, Lauren classification, Ming’s histological classification [83], stromal reaction type, and lymphovascular invasion pattern. The pathological tumor-node-metastasis (TNM) was evaluated by staging systems from the American Joint Committee on Cancer (AJCC) system [35].

These gastric cancer patients were followed up at the outpatient department every three months. Recurrence was defined as the first evidence of tumor relapse found by images, including upper GI endoscope, CT scan, or bone scan, and abdominal cytological analysis for ascites. The follow-up data were prospectively collected and regularly updated. The follow-up of these gastric cancer patients was continued for the maximum of 10 years or until death. Overall survival (OS) was defined from the date of surgery to the date of death or the date of the most recent follow-up date. Disease-free survival (DFS) was defined as the length of period after surgery during which the gastric cancer patient survived without recurrence.

### 4.3. Immunohistochemical (IHC) Staining Procedures and Interpretations

After surgery, specimens from 100 gastric cancer patients were selected for IHC staining using the previous protocol [82]. The primary antibody (Tid1 Cat number: sc-18819, Santa Cruz Biotechnology, Dallas, TX, USA) was used for IHC staining. The distribution of Tid-1 in gastric tumor specimens was calculated by a semi-quantitative system, which was evaluated by our expert gastrointestinal pathologist, Dr. Li AFY. The percentage of positive tumor expression and the arbitrary range were represented as low expression: −, <5% positive cells (trace) and 1+, 5–25% positive cells (weak); high expression: 2+, 26–75% positive cells (moderate) and 3+, more than 75% positive cells (strong).

### 4.4. Cell Culture

Human gastric cancer cells AGS, NUGC-3, and TSGH-9201 were purchased from the Bioresource Collection and Research Center (Food Industry Research and Development Institute, Hsinchu, Taiwan) and cultured in RPMI 1640 medium (Gibco, Grand Island, NY, USA) containing 10% fetal bovine serum (FBS, Gibco, Grand Island, NY, USA) and 1% antibiotic (penicillin-streptomycin, Biological Industries, Kibbutz Beit HaEmek, Israel), and incubated at 37 °C in a 5% CO_2_-containing incubator. When the cells were about 70−80% grown, sub-cultures were grown with PBS added to 1× Trypsin-EDTA (TE, Biological Industries, Kibbutz Beit HaEmek, Israel). The optimal passage ratios of these gastric cancer cells were 1:4–1:8.

### 4.5. siRNA Transfection

Tid1 gene knockdown was performed by small interfering RNA (siRNA) to knockdown *DNAJA3* (Tid1). A total of 2.0 × 10^5^ cells were seeded in a 6 cm dish. The siRNA mixed solution was prepared by dissolved lipofectamine RNAi MAX reagent (Thermo Fisher Scientific, Camarillo, CA, USA) and siRNA (60 pmol, ON-TARGET plus™ human DNAJA3 siRNA: Cat. No. L-017792-00-0010; non-target/scramble siRNAs: Cat. No. D-001810-01-05, GE Healthcare Dharmacon, Lafayette, CO, USA) in OPTI-MEM medium (Gibco™, Thermo Fisher Scientific, Grand Island, NY, USA) for 5 min at room temperature. The siRNA-lipid complex was dripped into the cell culture dish for transfection for 48 h.

### 4.6. Western Blot Analyses

The cellular protein was prepared by the collection of supernatant of RIPA lysis buffer after scraping and centrifugation at 13,000× *g* at 4 °C for 15 min. The protein concentration was analyzed by Bradford reagent (Bio-Rad, Hercules, CA, USA). The sample proteins (20–30 μg) were heated at 110 °C for 10 min and separated by electrophoresis (8–15% SDS-polyacrylamide gel electrophoresis). After protein separation, the proteins were transferred onto the polyvinylidene difluoride membranes (PVDF, Biotrace™, PALL Life sciences, Ann Arbor, MI, USA). Sample-PVDF sample membrane was blocked with 5% skimmed milk at room temperature for 1 h. The specific primary/secondary antibodies, enhanced chemiluminescence reaction, and luminescence fluorescence image capture system (GE healthcare) were used to observe specific proteins. The sample band was analyzed by MultiGauge image analysis software version 3.0 (Fujifilm, Stockholm, Sweden). The TID-1 L/S antibody (sc-18819) was obtained from Santa Cruz Biotechnology (Dallas, TX, USA). The galectin-7 antibody (Cat. No. PA5-31866) was obtained from Ambion™, Thermo Fisher Scientific (Eugene, OR, USA). The α-tubulin antibody was obtained from Invitrogen™, Thermo Fisher Scientific (Eugene, OR, USA). Chase analysis was performed by adding the cycloheximide (100 μg/mL) for the indicated period and analyzed by Western blot.

### 4.7. Sulforhodamine B (SRB) Cell Proliferation Assay and Chemotherapy Sensitivity Assay

In the SRB colorimetric assay, 3000 cells/well were seeded in a 96-well plate. Cells were cultured overnight and incubated or treated with drugs for the indicated times. At the indicated time, 10% trichloroacetic acid (TCA, Sigma-Aldrich, St. Louis, MO, USA) was used to fix the cells at 4 °C for 1 h. Then, the cells were washed with distilled water and air dried. Subsequently, cells were stained with 0.057% SRB (Sigma-Aldrich, St. Louis, MO, USA) at room temperature for 30 min and washed with 1% acetic acid (J.T. Baker, Center Valley, PA, USA). Finally, 10 mM Tris Base (J.T. Baker, Center Valley, PA, USA) was used to dissolve the SRB, and the absorbance value at a wavelength of 510 nm was determined using an ELISA reader. Cisplatin and 5-fluorouracil were obtained from Sigma-Aldrich (St. Louis, MO, USA). Doxorubicin and paclitaxel were obtained from Cayman chemical (Ann Arbor, MI, USA).

### 4.8. Soft Agar Colony Formation Assay

Sterile 3% agarose in PBS was used and mixed with FBS/cultured medium (1.05:0.45:4.5) to form the lower layer in a 6-well plate in an incubator for 1 h. After the lower layer was formed, 6 × 10^4^ cells/well with agarose/FBS/culture medium were added to the upper layer and incubated for 30 days. The colony was observed by microscope (40× magnification). Finally, five fields of view were photographed in each experimental group and quantitative analysis was performed using Image J software.

### 4.9. Tumor Sphere Formation Assay

A quantity of 1.0 × 10^3^ cells/well in 200 μL cultured medium were seeded in a non-attached 96-well culture plate. At the indicated time point, the number of spheres formed by the cells were photographed with a microscope. The microscope magnification was 40×. The sphere numbers (>100 μm) were counted using Image J software.

### 4.10. Wound Healing Migration Assay

A quantity of 8.0 × 10^5^ cells/well were seeded at 6 well plate. After cells were attached, 200 μL pipette micro tips were used to draw a cross in the well, and gently rinse twice with culture medium to remove floating cells. At the indicated time, cells were photographed with a microscope magnification of 40× and the migration distances were calculated using Image J software.

### 4.11. Transwell Migration Assay

1.0 × 10^5^ cells in a total volume of 200 μL serum-free medium were placed in a transwell insert (pore size of 8 μm). Subsequently, the inserts were transferred to a 700 μL (10% FBS-containing) cultured medium and to a 24-well plate in a 37 °C, 5% CO_2_ incubator and cultured for the indicated time periods. After the indicated time, the inserts were fixed by methanol at 4 °C for 20 min. Then, the inserts were stained with Liu’s stain A for 5 min/Liu’s stain B for 30 min at room temperature and washed with water. The inside of the insert was wiped and cleaned with a cotton swab. The cells were evaluated by microscope (magnification of 200×, five fields of view in each experimental group) and counted using Image J software.

### 4.12. Transwell Invasion Assay

The Matrigel Matrix was mixed with serum-free culture medium at a ratio of 1:6 (total volume 50 μL) and the Matrigel Matrix-medium mixture was added to a transwell insert (8 μm pore size) and incubated in 37 °C, 5% CO_2_ incubator for 1 h (condensing into a semi-solid state). A quantity of 1.0 × 10^5^ cells in a total volume of 150 μL serum-free medium was added to a matrix gel-containing insert for 48 h. Subsequently, the cells were fixed by methanol at 4 °C for 20 min and stained with Liu’s stain A/B (A: 5 min; B for 30 min) at room temperature. Inserts were washed with water and the inside was wiped clean with a cotton swab. After the inserts were air dried, the cells were analyzed using a Nikon ECLIPSE Ni-E Upright Motorized Microscope (magnification of 40×) and counted using Image J software.

### 4.13. mtDNA Copy Number Assay

MtDNA was extracted using QIAamp DNA Mini kit (Qiagen, Hilden, Germany). The primers for detecting the copy number of mtDNA were mtF3212, mtR3319, and 18S nDNA of nuclear DNA (nDNA, internal control). StepOne™ System (Applied Biosystems™ Real-time polymerase chain reaction (PCR) Instrument, Thermo Fisher Scientific) and KAPA SYBR FAST qPCR Kits (Kapa Biosystems, Wilmington, MA, USA) were used for real-time PCR reaction. The forward primer, mtF3212 5′-CACCCAAGAACAGGGTTTGT-3′, and the reverse primer, mtR3319 5′-TGGCCATGGGTATGTTGTTAA-3′, which were mapped to the sequence of the ND1 gene, were used to amplify a 108-bp PCR product for detection of the mtDNA copy number. The forward primer, 18s-1546F 5′-TAGAGGGACAAGTGGCGTTC-3′, and the reverse primer, 18s-1650R 5′-CGCTGAGCCAGTCAGTGT-3′ (mapped to the sequences of the 18S nDNA) were used to amplify a 105-bp product for internal control.

### 4.14. RNA Extraction and Quantitative Real-Time PCR

Cellular RNA extraction was performed using Trizol reagent according to its manual (Invitrogen™, Thermo Fisher Scientific, Carlsbad, CA, USA). The extracted 5 μg RNA was subjected to reverse transcription reaction using RevertAid™ reverse transcriptase (Thermo Fisher Scientific, Waltham, MA, USA). The obtained cDNA was used for real-time PCR reaction using StepOne™ System and KAPA SYBR FAST qPCR Kits. The real-time PCR reaction was denatured at 95 °C for 3 min, and the cycle conditions were 95 °C for 3 s and 60 °C for 30 s (40 cycles). The relative mRNA content was calculated using 2^−ΔΔCt^ with a GAPDH internal control. The primers for the indicated genes are listed in Table 2.

### 4.15. Analysis of Mitochondrial Mass and Mitochondrial Membrane Potential

The mitochondrial mass and mitochondrial membrane potential were analyzed by nonyl acridine orange (NAO) and JC-1 staining (Molecular Probe™, Invitrogen™, Thermo Fisher Scientific, Eugene, OR, USA), respectively. A quantity of 3.0 × 10^5^ cells was seeded in a 6-well plate and cultured overnight for attachment. Cells were collected and stained with 5 μM NAO at room temperature for 10 min or 1 mg/mL JC-1 at room temperature for 30 min. The mitochondrial mass and mitochondrial membrane potential were analyzed with a FACS Calibur flow cytometer (Becton Dickson). The fluorescence of NAO was measured using the FL-1 channel; the mitochondrial membrane potential was analyzed using the ratio of JC-1 fluorescence (FL-2/FL-1 channels). At least 15,000 cells were collected and analyzed in each sample using Cell Quest software (Becton Dickinsin).

### 4.16. Mitochondrial Oxygen Consumption (OCR) Assay

OCR was measured using a XF24 extracellular flux analyzer (Seahorse Bioscience, Agilent, Santa Clara, CA, USA). During the experiment, oligomycin was used to determine the ATP-link OCR, CCCP was used to determine the maximal OCR, and antimycin A was used to exclude the mitochondria-independent oxygen consumption. Each cycle of measurement consisted of 3 min mixing, 2 min waiting and 3 min measuring. OCR was normalized to the cell number at the end of the experiments by SRB assay. To obtain the mitochondrial-dependent OCR, only the antimycin sensitive respiration was analyzed.

### 4.17. Intracellular and Mitochondrial ROS Analyses

Cellular ROS and mitochondrial ROS were detected with dichlorodihydro-fluorescein diacetate (DCFH-dA) and MitoSOX Red, respectively. A quantity of 2 × 10^5^ cells/well was seeded in a 6-well plate. After attachment, cultured medium was replaced with fresh medium containing 5 μM DCFH-dA at 37 °C for 30 min or containing 10 μM MitoSOX Red at 37 °C for 10 min. Cell pellets were resuspended with PBS and analyzed by flow cytometry. The DCFH-dA and MitoSOX Red (obtained from Molecular Probes™, Invitrogen, and Thermo Fisher Scientific, Eugene, OR, USA) fluorescent intensities were detected using FL1 and FL2 detectors, respectively. A minimum of 15,000 cells were analyzed in each sample using flow cytometry and Cell Quest software.

### 4.18. Gelatin Zymography

A quantity of 7.0 × 10^5^ cells was seeded in a 6 cm dish and the cultured medium with serum-free medium was collected for 40 h. The supernatant of the collected culture medium was centrifuged at 4 °C 12,000× *g* for 10 min. The 5–10 μg samples were mixed with 5× sample dye (non-reducing), and electrophoresis separation was performed at 150 V (SDS-PAGE with 2% gelatin in the gel, running buffer: Tris Base, glycine, and 0.1% SDS). After washing the gel with washing buffer (containing Triton X-100, Tris-HCl, CaCl_2_, and ZnCl_2_), the gel was incubated in a 37 °C incubator for 17–24 h with incubation buffer (containing Triton X-100, Tris-HCl, CaCl_2_, and ZnCl_2_). The gel was stained with staining buffer (containing methanol, acetic acid, and Coomassie Blue) and persistently destained with destain buffer (containing methanol and acetic acid) until the white band was visualized. The band was analyzed using an Amersham Imager 680 blot and gel imager (GE healthcare) and MultiGauge image analysis software version 3.0 (Fujifilm, Stockholm, Sweden).

### 4.19. Statistical Analyses

The data obtained in the presented experiments were expressed as means ± SD and analyzed using the Student’s *t* test. A *p*-value less than 0.05 indicated a significant statistical difference. The analysis was performed using GraphPad PRISM software, version 6 (La Jolla, CA, USA). The statistical analyses of the enrolled gastric tumor specimens were calculated using SPSS software version 20.0 (SPSS Inc., Chicago, IL, USA). Clinical data and pathological differences of gastric cancer patients were estimated using chi-squared tests. Multivariate analyses were carried out using Cox logistic regression models. The OS and DFS were calculated using a log rank test and Kaplan-Meier survival curve analysis. We defined a statistically significant *p*-value as less than 0.05.

## 5. Conclusions

In summary, the present study provided the clinical correlations of Tid1 gene and protein expressions in gastric cancer patients. Low Tid1 protein expression might be associated with the high extent of lymph node invasion and poor prognosis of gastric cancer patients. Moreover, knockdown of Tid1 enhanced cell migration and invasion of gastric cancer cells. In addition, Tid1 knockdown reduced the mitochondrial mtDNA copy number. These results suggest that Tid1, a mitochondrial co-chaperone, might be required for mtDNA maintenance and to regulate migration and invasion of gastric cancer cells, which may contribute to lymph node invasion and poor prognoses in gastric cancer patients. Our findings may provide a potential target for development of future gastric cancer therapies.

## Figures and Tables

**Table 1:** Clinical profiles of 100 gastric cancer patients with low or high Tid1 expression in tumors.

Characteristic	Tid1 Low Expression (0,1) (*n* = 30)	Tid1 High Expression (2,3) (*n* = 70)	*p*
Age (mean ± SD)	67.80 ± 12.0	67.49 ± 12.6	0.908
Gender			
Male	22	57	
Female	8	13	0.362
Tumor size			
<4 cm	5	19	
4–8 cm	17	38	
>8 cm	8	13	0.441
Tumor location			
Upper stomach	5	15	
Middle stomach	15	25	
Lower stomach	10	27	
Whole stomach	0	3	0.548
Cell grade			
Poor differentiated	18	35	
Moderate differentiated	11	34	
Well differentiated	1	1	0.381
Gross appearance			
Superficial type	1	0	
Borrmann type I & II	7	25	
Borrmann type III & IV	22	45	0.177
Stromal reaction type			
Medullary type	2	4	
Intermediate type	15	51	
Scirrhous type	13	15	0.058
Lauren’s histology			
Intestinal type	8	27	
Diffuse type	14	19	
Mixed type	8	24	0.161
Ming’s histology			
Expanding	2	7	
Infiltrating	28	63	0.720
Lymphovascular invasion			
No	8	19	
Yes	22	51	0.133
TNM pathological T category			
T1	10	35	
T2	7	15	
T3	11	14	
T4	2	6	0.289
TNM pathological N category			
N0	4	19	
N1	5	20	
N2	7	17	
N3	14	14	0.041 *
TNM Stage			
I	1	9	
II	6	18	
III	23	43	0.274
Overall survival rate (5 years)	43.3%	58.5%	0.082
Disease-free survival rate (5 years)	36.7%	57.1%	0.008 *

*, *p* < 0.05, statistical significance. #, American Joint Committee on Cancer (AJCC) Cancer Staging Manual, eighth edition, T category: T1, T2, T3, T4; N category: N0, N1, N2, N3.

**Table 2:** The real-time PCR primers used.

Gene	Forward Primer Sequence	Reverse Primer Sequence
*GAPDH*	5′-CCGTCTAGAAAAACCTGCC-3′	5′-GCCAAATTCGTTGTCATACC-3′
*Tid1-L*	5′-GTTCCAAAGAGGCTAACGAG-3′	5′-TTGCTTCCTGCGGAGCTATC-3
*Tid1-S*	5′-GTTCCAAAGAGGCTAACGAG-3′	5′-GGCCTAGTTTCCAGTGGATC-3′
*mt-ND1*	5′-CCCTAAAACCCGCCACATCT-3′	5′-GGCTAGAATAAATAGGAGGCCTAGGT-3′
*mt-ND2*	5′-AACCCGTCATCTACTCTACCATCT-3′	5′-GCTTCTGTGGAACGAGGGTTTATTT-3′
*mt-ND3*	5′-CCACAACTCAACGGCTACATAGAAA-3′	5′-GGGTAAAAGGAGGGCAATTTCTAGA-3′
*mt-ND4*	5′-TCACAACACCCTAGGCTCACTAA-3′	5′-GGGAGTCATAAGTGGAGTCCGT-3′
*mt-CYB*	5′-TCACCTCCCATTCCGATAAAATCAC-3′	5′-GGGTTGGCTAGGGTATAATTGTCTG-3′
*mt-CO1*	5′-CAGCAGTCCTACTTCTCCTATCTCT-3′	5′-GGGTCGAAGAAGGTGGTGTT-3′
*mt-CO2*	5′-GGGTCGAAGAAGGTGGTGTT-3	5′-GTAAAGGATGCGTAGGGATGGG-3′
*mt-CO3*	5′-TCACCTGAGCTCACCATAGTCTAAT-3′	5′-GCCGTCGGAAATGGTGAAG-3′
*mt-ATP*	5′-CGTACGCCTAACCGCTAACATT-3′	5 -GCGACAGCGATTTCTAGGATAGT-3′
*Snail*	5′-ACATCCGAAGCCACACGCTGC-3′	5′-CGCAGGTTGGAGCGGTCAGC-3′
*Slu*	5′-TGTGTGGACTACCGCTGC-3′	5′-TCCGGAAAGAGGAGAGAGG-3′
*Vimentin*	5′-CCTTGAACGCAAAGTGGAATC-3′	5′-GACATGCTGTTCCTGAATCTGAG-3′
*β-catenin*	5′-CCAGCCGACACCAAGAAG-3′	5′-CGAATCAATCCAACAGTAGCC-3′
*E-cadherin*	5′-CCCACCACGTACAAGGGTC-3′	5′-CTGGGGTATTGGGGGCATC-3′
*ZEB1*	5’- TTCAAACCCATAGTGGTTGCT-3’	5’- TGGGAGATACCAAACCAACTG-3’
*IL-8*	5′-AATTGAGGCCAAGGGCCAAGAG-3′	5′-AGGACTTGTGGATCCTGGCTAG-3′
*MMP-9*	5′-TTGACAGCGACAAGAAGTGG-3′	5′-CCCTCAGTGAAGCGGTACAT-3′

## Supplementary Materials

The following are available online at https://www.mdpi.com/2072-6694/12/11/3463/s1, Figure S1: Full blots corresponding to Figure 2, Figure S2: Full blots corresponding to Figure 3A, Figure S3: Full blots corresponding to Figure 3B, Figure S4: Full blots corresponding to Figure 3C, Figure S5: Full blots corresponding to Figure 4, Figure S6: Full blots corresponding to Figure 5A, Figure S7: Full blots corresponding to Figure 5B, Figure S8: Full blots corresponding to Figure 6, Figure S9: Full blots corresponding to Figure 7A, Figure S10: Full blots corresponding to Figure 7B, Figure S11: Full blots corresponding to Figure 7C.

## Abbreviations

ATP	Adenosine triphosphate
ROS	reactive oxygen species
OXPHOS	oxidative phosphorylation
mtDNA	mitochondrial DNA
Tid1	Tumorous imaginal disc 1
DNAJA3	DnaJ homolog subfamily A member 3
Hsp	heat shock protein
AJCC	American Joint Committee on Cancer
DFS	disease free survival
OS	overall survival
SRB	sulforhodamine B
OCR	oxygen consumption rate
MMP-9	matrix metallopeptidase 9
EMT	epithelial to mesenchymal transition
IL-8	interleukin-8
HER2	human epidermal growth factor receptor 2
STAD	stomach adenocarcinoma
CT	computed tomography
TNM	tumor-node-metastasis
IHC	immunohistochemical
PVDF	polyvinylidene difluoride membranes
NAO	nonyl acridine orange
DCFH-dA	dichlorodihydro-fluorescein diacetate
EGFR	epidermal growth factor receptor
