# Supplementary material for: DNAJA3/Tid1 Is Required for Mitochondrial DNA Maintenance and Regulates Migration and Invasion of Human Gastric Cancer Cells
Source: Cancers (Basel). 2020 Nov 20;12(11):3463. doi: 10.3390/cancers12113463 (PMC7699785; doi:10.3390/cancers12113463)
Supplement: Supplementary file 1 [file cancers-12-03463-s001.pdf]

cancers

Supplementary Materials: DNAJA3/Tid1 Is Required for Mitochondrial DNA Maintenance and Regulates Migration and Invasion of Human Gastric Cancer Cells

Sheng-Fan Wang, Kuo-Hung Huang, Wei-Chuan Tseng, Jeng-Fan Lo, Anna Fen-Yau Li, Wen-Liang Fang, Chian-Feng Chen, Tien-Shun Yeh, Yuh-Lih Chang, Yueh-Ching Chou, Hung-Hsu Hung and Hsin-Chen Lee

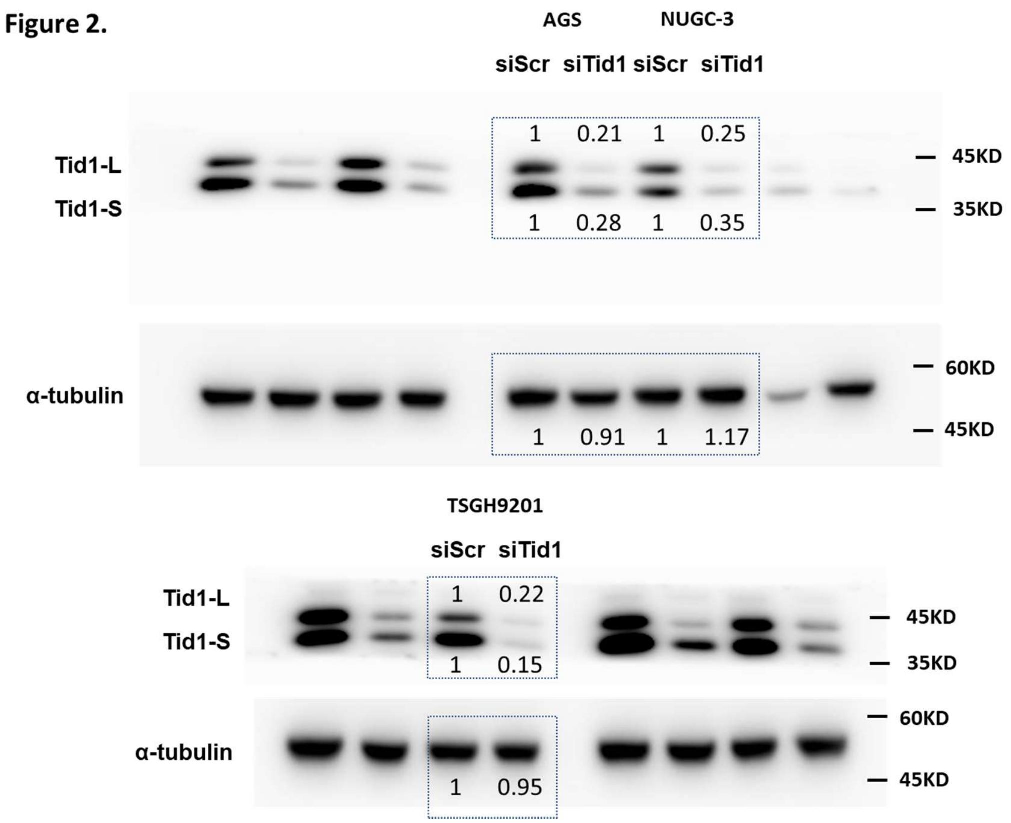

Figure S1. Full blots corresponding to Figure 2.

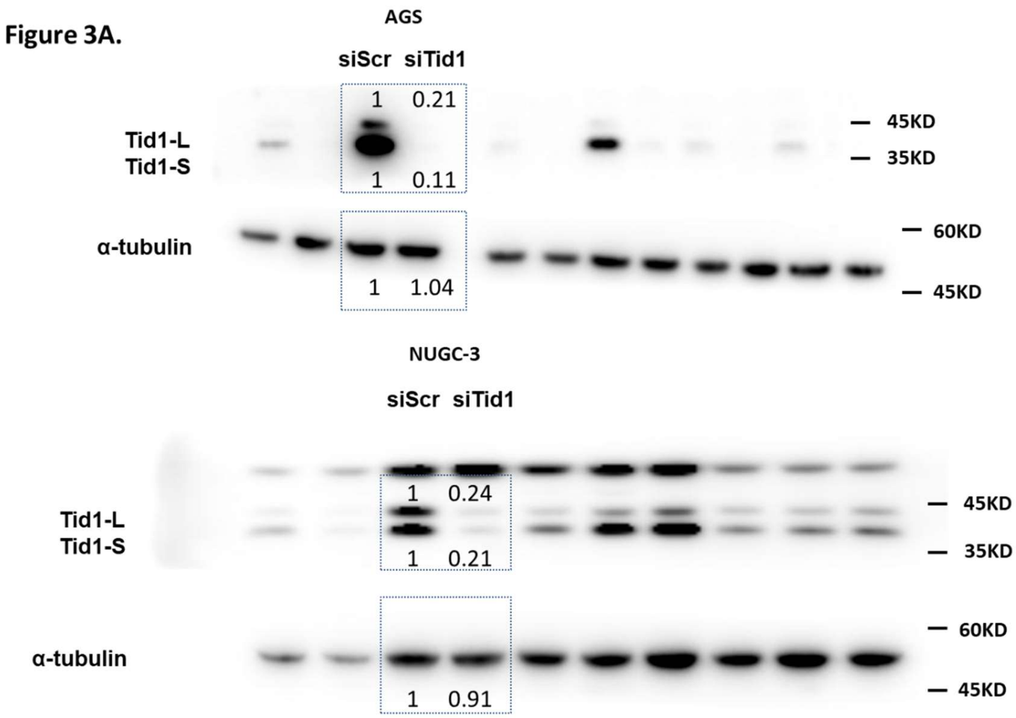

Figure S2. Full blots corresponding to Figure 3A.

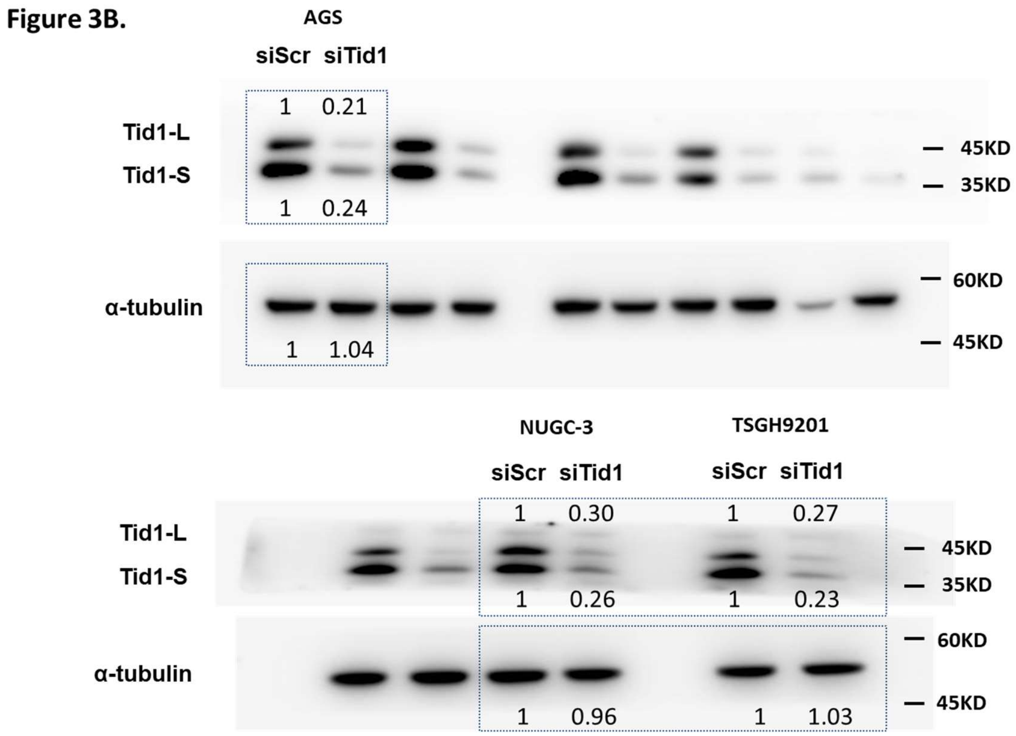

Figure S3. Full blots corresponding to Figure 3B.

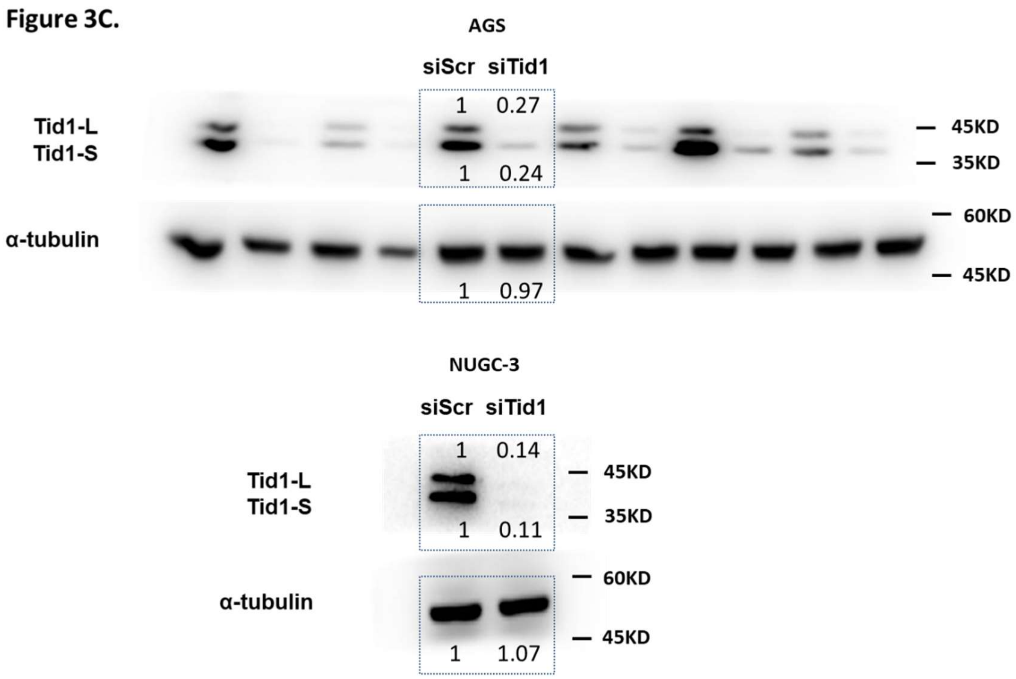

Figure S4. Full blots corresponding to Figure 3C.

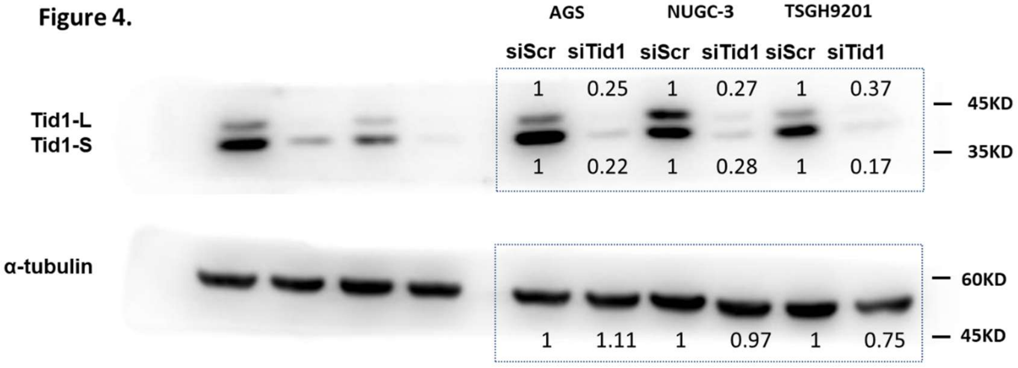

Figure S5. Full blots corresponding to Figure 4.

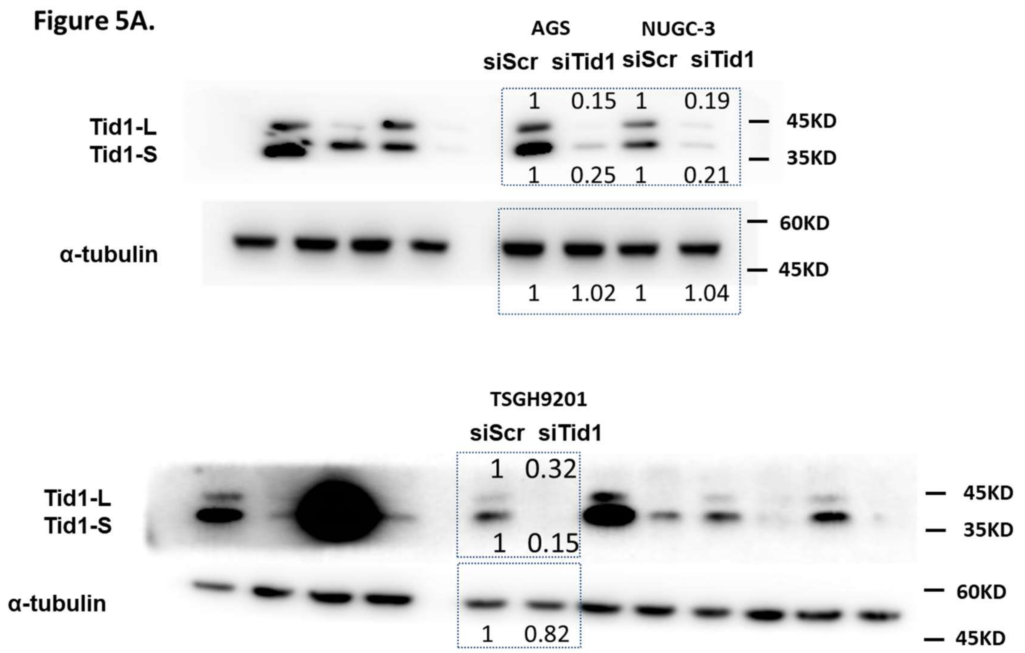

Figure S6. Full blots corresponding to Figure 5A.

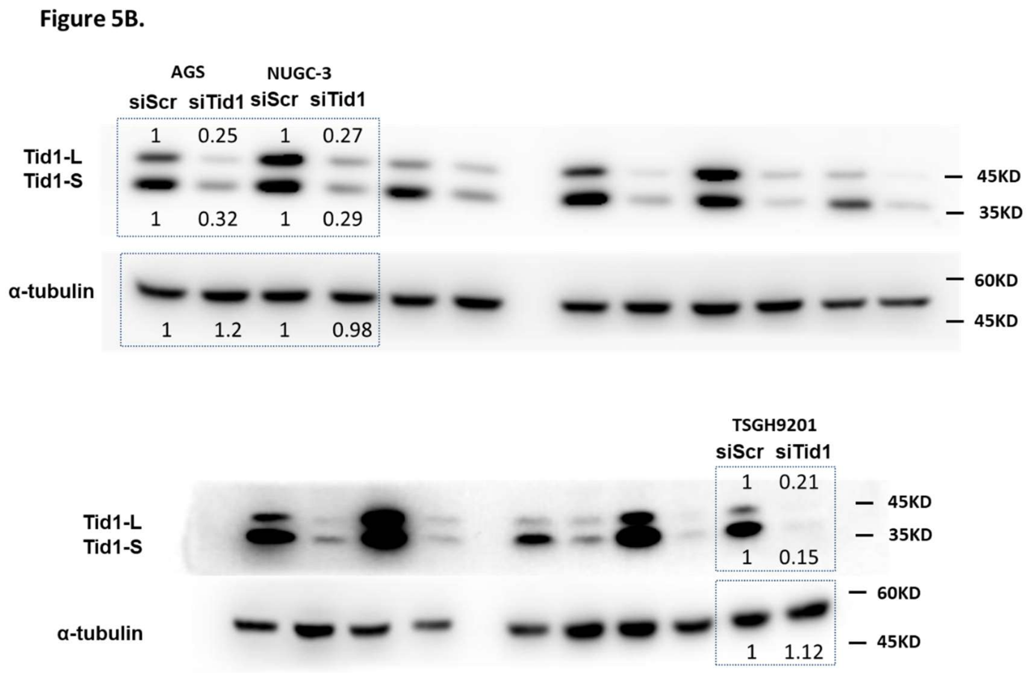

Figure S7. Full blots corresponding to Figure 5B.

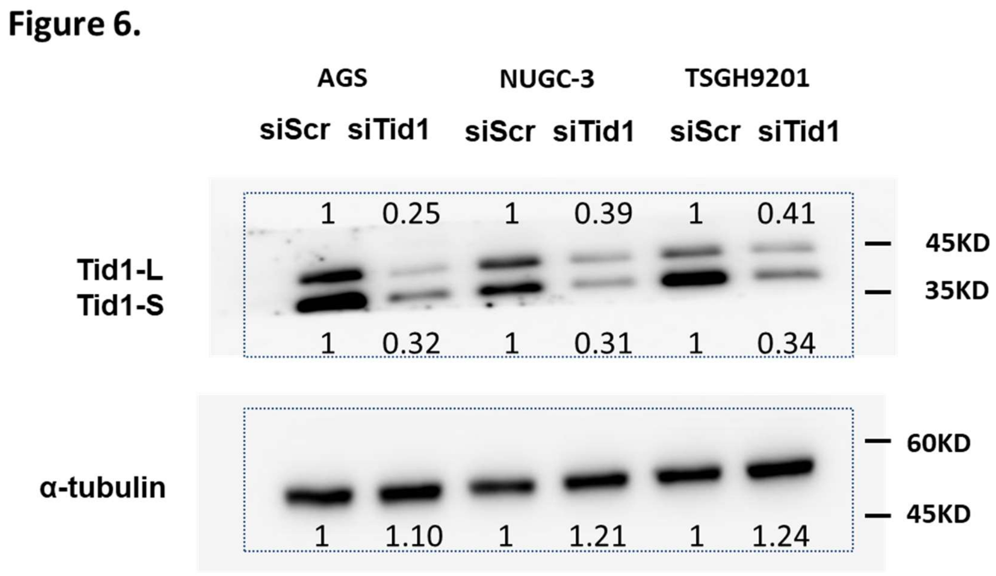

Figure S8. Full blots corresponding to Figure 6.

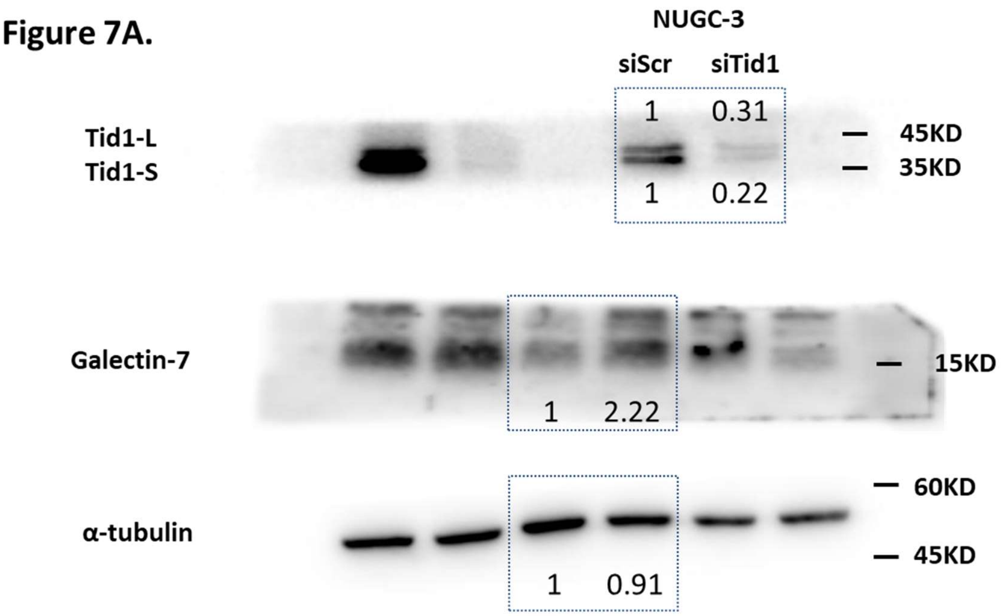

Figure S9. Full blots corresponding to Figure 7A.

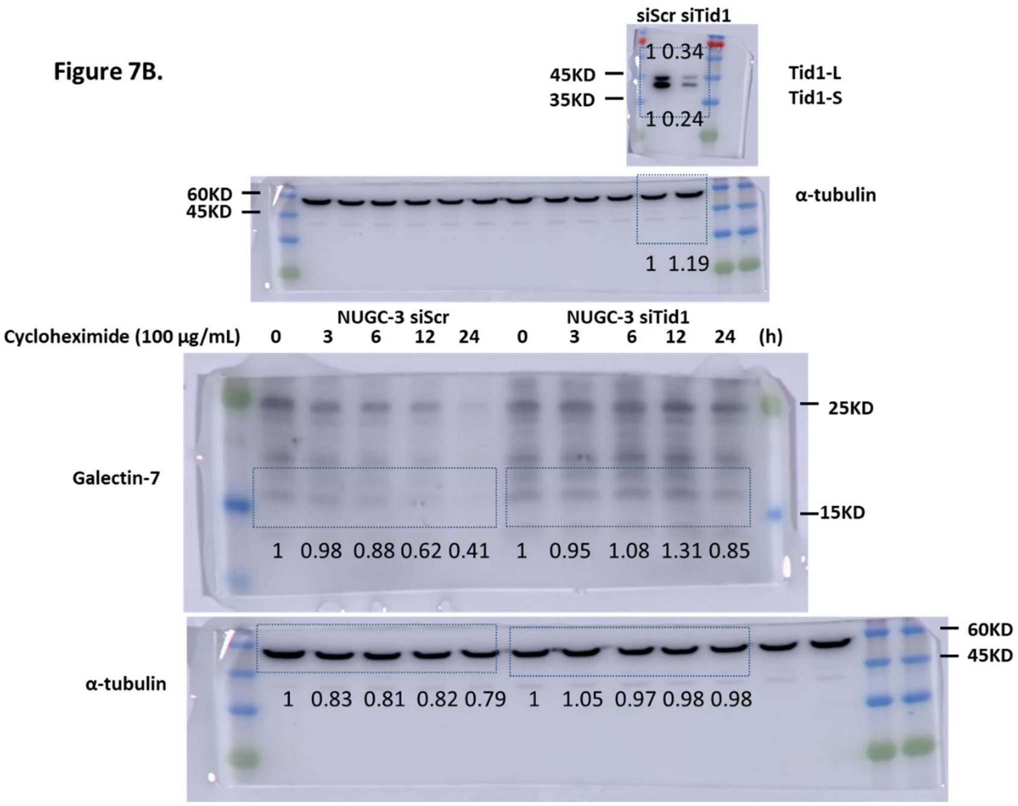

Figure S10. Full blots corresponding to Figure 7B.

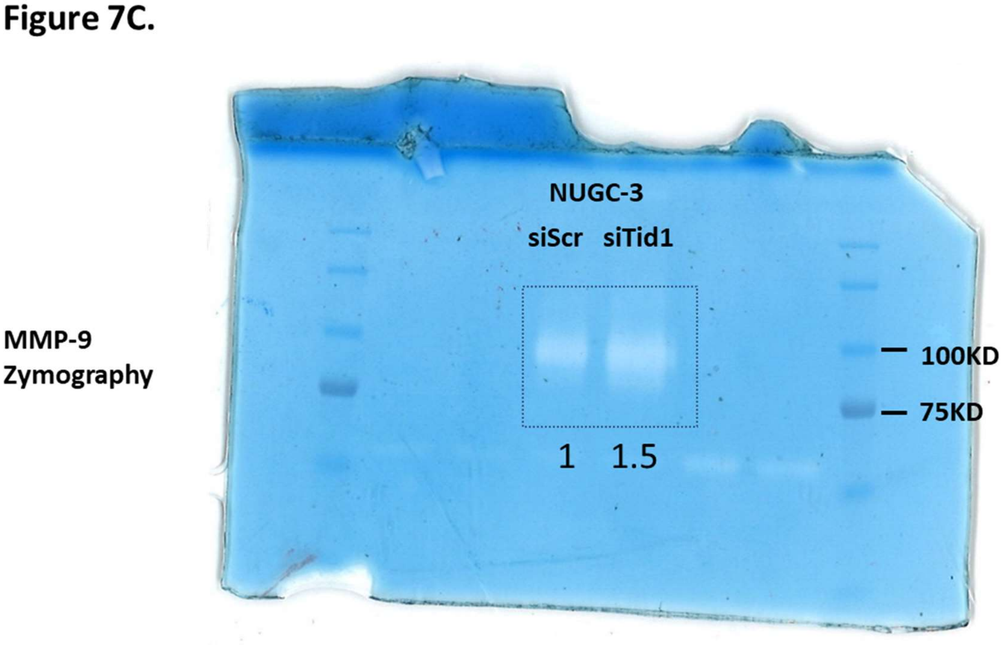

Figure S11. Full blots corresponding to Figure 7C.
